# Editorial: Natural products and brain energy metabolism: Astrocytes in neurodegenerative diseases

**DOI:** 10.3389/fphar.2022.1039904

**Published:** 2022-10-03

**Authors:** Fushun Wang, Shijun Xu, Fang Pan, Alex Verkhratsky, J. H Huang

**Affiliations:** ^1^ Institute of Brain and Psychological Science, Sichuan Normal University, Chengdu, China; ^2^ School of Pharmacy, Chengdu University of Traditional Chinese Medicine, Chengdu, China; ^3^ Department of Medical Psychology, Shandong University Medical School, Jinan, China; ^4^ Department of Physiology, The University of Manchester, Manchester, United Kingdom; ^5^ Department of Neurosurgery, Baylor Scott & White Health, Temple, TX, United States; ^6^ Department of Surgery, Texas A&M University College of Medicine, Temple, TX, United States

**Keywords:** astrocyte, mitochondria, energy supply, glymphatic, emotinoal arousal, monoamine

The brain weighs approximately 2% of the body, but utilizes about 20% of the total energy and oxygen supply, thus energy supply is critically important for the brain. In addition, the brain has no energy reserve, except some glycogens in the astrocytes, and the neurons can only use ATP, which comes from glucose degradation in the essential structure mitochondria. Mitochondria impairment, such as electron transport chain damage, can induce decreased ATP levels, decreased antioxidant defense, which is prevalent in many neurodegenerative disorders. For example, ATP dysfunction has been suggested to be the major impaired in depression (Cao, et al., 2013). Numerous studies suggest that energy metabolism disorders are hallmarks of brain aging and are particularly involved in many neurodegenerative disorders, such as Alzheimer’s diseases and major depression disorder. However, even though the regulatory mechanisms are important to ensure adequate supply for the neurons, how the brain accomplishes this complex task is still unclear.

## Emotional arousal regulates brain energy supply

It is critically important to understand the central facets of physiology and pathophysiology of energy supply in the brain, however, the energy supply regulation in the brain is not clear, for example how emotion affects energy supply, and does it activated by monoamine neurotransmitters. Emotion plays an important role in the regulation of the energy supply for the brain. Emotion parallels with behavior all the time, from the initiation of a behavior, the process of behavior to the end of the behavior. Emotion is the tendency for behavior, is also the motivation of a behavior. Emotion is also important for appraisal of behavioral consequences, marking the behavior as reward or punishment (Gu, et al., 2019). Emotions have two dimensions: arousal and valence. Emotional arousal can activate the sympathetic nervous system to increase the heart beat and breath rate to increase blood supply, including glucose and oxygen for the brain and induced “fight or flight” behaviors (Liang et al., 2021). Emotional valence is related to physiological needs and can activate the parasympathetic nervous system to increase activities of digestive system, and also increase breath depth (Zheng et al., 2016).

Emotion is nothing but neurotransmitters (Gu, et al., 2019). The emotional neurotransmitters not only affect the periphery organs *via* autonomous nervous system, but also affect the energy supply in the brain. For example, the monoamine transmitters stimulate Na^+^, K^+^-ATPase activity in astrocytes and facilitate their role in ion concentrations (Wang et al., 2012, 2013). Previously, we proposed a “three primary color model of basic emotions, which suggested that the emotions are composed of three primary emotions: joy, fear (anger) and disgust (Wang et al., 2020, 2022). The three primary emotions depend on three monoamines: Joy-dopamine (DA), fear (anger)- norepinephrine (NE) and disgust-serotonin (5-HT) (He et al., 2021). The relationship of cerebral neurotransmitters DA, NE, 5-HT to the energy state of the brain has been investigated by many studies, for example, it is found that many environmental and homeostatic challenges can induce monoamine release, and induce rapid preparatory changes in neural activities as well as increased brain energy metabolism (Beley et al., 1991).

## Astrocytes in energy supply for the brain

Blood glucose is the major source of energy supply for the brain, which can be taken up by both neurons and astrocytes, however, astrocyte has been known to play a major support role in energy supply in the brain. “Glycogenetic” hypothesis suggests that astrocyte plays a more important role in glycolytic metabolism, while neurons function as high mitochondrial oxidative activity to use energy. Glycogen reservoir in astrocytes can be used as brain energy supply by turning glycogen to glucose and release glucose at high extracellular K^+^ and glutamate release during brain arousal (Petit et al., 2021).

Monoamine neurotransmitters are exquisitely suitable for regulation of astrocytic glucose uptake and release. Astrocytes can turn glycogen to glucose at emergent energy demand, when monoamine neurotransmitters are released at emotional arousal. Emotional arousal can induce NE release in the locus coeruleus and also other monoamine neurotransmitters, which are glycogenolytic agents (DiNuzzo et al., 2015). Emotional arousal induced activation of noradrenergic locus coeruleus can reversibly block astrocytic glucose uptake by glycogen mobilization in astrocytes, possibly *via* Ca^2+^ signaling in astrocytes (Ding, et al., 2013). Accumulating evidence indicates that emotions can affect energy metabolism *via* astrocytic Ca^2+^ signaling, which can affect the glucose, ions and also gliotransmitter release. Previously, we have found that NE induced *via* astrocytic Ca^2+^ signaling can affect extracellular K^+^ (Wang et al., 2012). In addition, these astrocytic Ca^2+^ signaling can also affect the function of mitochondria, whose normal functions rely on Ca^2+^. Indeed, chronic stresses have been found to induce mitochondrial abnormalities and cause energy shortage in neurons (Iwata et al., 2019). In addition, energy supply shortage is a major cause for a cascade of neurosynaptic dysfunctions, which leads to Alzheimer’s disease, Parkinson disease, stress, and depression etc*.*

## Astrocytes in clearance of metabolic waste

Astrocyte is known as an energy support system for the brain, and also a clearance system to clear off the metabolic waste in the brain microenvironment. The Glymphatic system is a unique clearance system in the brain to extrude water and macromolecular waste (Mestre et al., 2020). The glymphatic system is a drainage system that is composed of perivascular spaces and astrocytes to connect the brain interstitial fluids and cerebrospinal fluid (Iliff, et al., 2013). These fluids flow along the paravascular space of the arteries and veins to essentially clear off the metabolites. The function of glymphamatic system is controlled by monoamine neurotransmitters, especially NE, which can block the function of glymphamatic system by modulating astrocytic functions (Ding et al., 2016). Thus sleep is very important for normal function of glymphamatic system to effectively control the clearance of toxic peptides such as tau protein.

The glymphatic system, which was found in our lab in 2012, has recently been found to play an important player in many neurodegenerative diseases, because of vasculature impairment, or astrocytic dysfunction. The glymphatic system is a brain-wide perivascular pathway that is potentially offering new therapeutic targets to improve cerebral drainage and immune survey in human CNS diseases (Ma et al., 2021). Indeed, many genetic and pharmacological approaches have proved that “housekeeping” effects of astrocytes are involved in many neurodegenerative diseases by supplying energy and clearance of metabolites.

## Natural product for energy supply

Many natural plants have been used to treat the neurodegenerative disorders in Traditional Chinese Medicine or other alternative treatments. Some natural products have been identified and proved to be effective in treating the neurodegenerative disorder, such as flavonoids, polyphenols, alkaloids. The discovery of strong active compounds from plants has been validated for efficacy, and structural modification based on the groups is an effective method for new drug discovery. For example, Huperzine A from Huperzia serrata is proven to improve memory impairment in Alzheimer’s disease, and huperzine A-derived Shiplin is currently in Phase III clinical studies. This lesson could reveal a new opportunity for natural compounds as candidates for neurodegenerative diseases therapy.

## This topic collected papers

In this Research Topic, we welcomed high-quality studies about natural products regulating energy metabolism at neurodegenerative diseases. We got 35 submissions and accepted 15 paper *via* peer-reviewed processes, all the accepted papers are introduced below:

In the paper titled “Curcumin as a holistic treatment for tau pathology”, the author Sivanantharajah reviewed recent studies about traditional herbs, spices and other nutraceuticals that can be used to effectively treat AD. They found that the spice Turmeric with its active ingredient curcumin can effectively be used to treat tau pathology.

In the experimental study titled “Tanhuo Formula Inhibits Astrocyte Activation and Apoptosis in Acute Ischemic Stroke”, Nie et al. introduced an old Chinese formula Tanhuo formula. They first analyzed its bioactive compounds and then tested their effects on the activities of astrocytes, and found that Tanhuo formula can effectively block neural cell apoptosis *via* caspase-3 pathway to block the excessive abnormal and the release of TNF-α and IL-6.

In the review titled “Berberine: a promising treatment for neurodegenerative diseases”, the authors Cheng et al., gave a short review about berberine, which is alkaloid compound from a kind of Chinese herb. Many recent studies have reported the effects of berberine on neurodegenerative disorders, such as Parkinson’s disease, Alzheimer’s disease, Huntington’s disease and so on. The review suggested that the major function of berberine is inhibiting oxidative stress and endoplasmic reticulum stress, enhancing mitochondria activity, which can in turn induce neuronal damage and apoptosis.

In another paper titled “Current evidence and future directions of berberine intervention in depression”, Zhu et al. also reviewed the effects of berberine, natural monomer compound of Coptis chinensis, and its effects on the typical emotional disorder (major depressive disorders), and suggested the berberine might be used as encouraging antidepressants to modulate depressed emotion.

In the experimental study about berberine titled “Berberine Alleviate Cisplatin-induced Peripheral Neuropathy by Modulating Inflammation Signal *via* TRPV1”, the authors Meng et al. studied the effects of berberine on transient receptor potential vanilloid (TRPV1) in dorsal root ganglia inflammation *via* inhibiting NF-κB and activating the JNK/p38 MAPK pathways in early injury, which inhibited the overexpression of TRPV1. The study suggested that berberine can reverse neuropathic pain response *via* inhibiting TRPV1 expression.

In another review, the authors Li et al. introduced another kind of natural product, echinacoside, which is a kind of phenylethanoid glycoside (PhG) in Cistanche tubulosa. In the paper “Therapeutic potential and molecular mechanisms of Echinacoside in neurodegenerative diseases”, they reviewed many recent studies about its mechanisms in neuroprotective efficacy in the prevention and treatment of neurodegenerative diseases, and proposed that the major effect of echinacoside is improving mitochondrial function and reducing anti-oxidative stress.

In the review paper titled “Progress in the mechanism of autophagy and traditional Chinese medicine herb involved in dementia”, Tao et al. summarized recent *in vitro* and *in vivo* studies about Chinese herbs in treating dementia, and found that the extracts of these Chinese herbs work on reducing generation of reactive oxygen species and inhibiting inflammation and neurotoxicity.

In the experimental paper titled “Yi-zhi-fang-dai formula exerts neuroprotective effects against pyroptosis and blood-brain barrier-glymphatic dysfunctions to prevent amyloid-beta ccute accumulation after cerebral ischemia and reperfusion in rats”, the authors Lyu et al. studied a kind of Chinese herb Yi-zhi-fang-dai formula, and its effects on pyroptosis and glymphatic dysfunctions. The study suggested that the Chinese herb Yi-zhi-fang-dai formula could inactivate pyroptosis *via* inhibiting caspase-1/11 activation and gasdermin D cleavage, and induce AQP-4 depolarization thus increase glymphatic function to reduce neuronal damage.

In the experimental paper titled “Rhein relieves oxidative stress in an Aβ1-42 oligomer-burdened neuron model by activating the SIRT1/PGC-1α-regulated mitochondrial biogenesis”, the authors Yin et al. studied the antioxidant activity of natural product rhein, and also its effects in clearance of β-amyloid (Aβ) in Alzheimer’s disease. The study found that rhein could significantly reduce reactive oxygen species, and activate mitochondrial biogenesis by increased cytochrome C oxidase and superoxide dismutase activities.

In the experimental paper “Neuronal Regeneration by Downregulating Notch Signaling Pathway in the Treatment of Generalized Anxiety Disorder”, the authors Liu et al. introduced a study about a kind of Chinese herb named danzhi xiaoyao powder and its effects on the symptoms of generalized anxiety disorder. The results suggested that the power could improve the weight growth and improve appetite and reduce the anxious mood *via* notch signaling pathway in the hippocampus. The study suggested that danzhi xiaoyao powder is a good therapy method for mood disorders.

In the experimental study, He et al. introduced a kind of classic Chinese herb formula, qiangji decoction in the paper “Qiangji Decoction alleviates neurodegenerative changes and hippocampal neuron apoptosis induced by d-galactose *via* regulating AMPK/SIRT1/NF-κB signaling pathway”. The herbs have been widely used in the traditional Chinese medicine, but the mechanisms are not clear. This paper found the major effect of qiangji decoction is reducing inflammation *via* reducing TNF-α, IL-1β and IL-6 in the hippocampus *via* regulating AMPK/SIRT1/NF-κB signaling pathway in hippocampal neuron apoptosis.

In the experimental study, Zheng et al. introduced a kind of Chinese herb ginkgo biloba extract and donepezil on Alzheimer’s disease. In the paper titled “Effects of ginkgo biloba extract and donepezil on functional recovery in Alzheimer’s disease: a multi-level characterized study based on clinical features and resting-state functional magnetic resonance imaging”, they used neuroimage study and found that the herbs could change the ALFF values in right gyrus rectus and decreased PerAF values in left fusiform gyrus. And the authors concluded that the imaging metrics in specific brain regions may serve as biomarkers for therapeutic efficacy of medicines.

The authors Zheng et al. introduced a study on the mechanism of sytisine on temporal lobe epilepsy, in the paper titiled “Cytisine exerts an anti-epileptic effect *via* α7nAChRs in a rat model of temporal lobe epilepsy”. Cytisine is an agonist of α7 nicotinic acetylcholine receptors (α7nAChRs) and has shown neuroprotection in many neurological diseases. This study found that sytisine could increase hippocampal function *via* enhancing ACh levels and α7nAChR expression, and decrease glutamate level to reduce seizures. The authors Arrodi et al. introduced one paper titled “Modulatory effects of alpha- and gamma-tocopherol on the mitochondrial respiratory capacity and membrane potential in Alzheimer’s disease an *in vitro* model of Alzheimer’s disease”, which suggested that mitochondrial abnormalities are an early feature in the pathogenesis of AD. They found that alpha-tocopherol or gamma-tocopherol could modulate mitochondrial function by increasing the production of ATP and reducing mitochondrial reactive oxygen species *via* altering mitochondrial metabolic pathways such as oxidative phosphorylation.

In the paper titled “Tortoise plastron and dear antler gelatin prevents against neuronal mitochondrial dysfunction *in vitro*: implication for a potential therapy of Alzheimer’s disease”, Cheng et al. investigated the role of mitochondrial dysfunction in the pathogenesis of Alzheimer’s disease. In addition, they investigated the effects of tortoise plastron gelatin and dear antler gelatin in preventing neuronal mitochondria function. The found that these two natural products could increase cell viability by modulating intracellular ATP and calcium level, and also regulate mitochondrial membrane potential (MMP) and ultrastructure, and finally inducing anti-dementia effects.

In all, these studies demonstrate that astrocytic function and neuronal mitochondria function play important roles in many neurodegenerative disorders. In addition, this topic introduced many natural drugs in treating these diseases. We expected that this Research Topic will stimulate interest in the study of the mechanisms of modulating the energy supply.
